# Assessment of Olfactory Threshold in Patients Undergoing Radiotherapy for Head and Neck Malignancies

**Published:** 2014-10

**Authors:** Mir Mohammad Jalali, Hooshang Gerami, Abbas Rahimi, Manizheh Jafari

**Affiliations:** 1 *Department of Otorhinolaryngology, Amiralmomenin Hospital, Guilan University of Medical Sciences, Guilan, Iran. *; 2 *Department of Radiotherapy, Razi Hospital, Guilan University of Medical Sciences, Guilan, Iran.*

**Keywords:** Olfactory perception, Radiotherapy, Thermoluminescent dosimetry

## Abstract

**Introduction:**

Radiotherapy is a common treatment modality for patients with head and neck malignancies. As the nose lies within the field of radiotherapy of the head and neck, the olfactory fibers and olfactory receptors may be affected by radiation. The aim of this study was to evaluate changes in olfactory threshold in patients with head and neck malignancies who have received radiation to the head and neck.

**Materials and Methods:**

The olfactory threshold of patients with head and neck malignancies was assessed prospectively before radiation therapy and serially for up to 6 months after radiotherapy using sniff bottles. In vivo dosimetry was performed using 82 LiF (MCP) chips and a thermoluminescent dosimeter (TLD) system.

**Results:**

Sixty-one patients were recruited before radiotherapy was commenced. Seven patients did not return for evaluation after radiation. Fifty-four patients were available for follow-up assessment (28 women, 26 men; age, 22–86 years; median, 49 years). Total radiation dose was 50.1 Gy (range, 30–66 Gy). Mean olfactory threshold scores were found to deteriorate significantly at various timepoints after radiotherapy (11.7 before radiotherapy versus 4.0 at Month 6, general linear model, P<0.0001). With in vivo dosimetry, we found that the median measured dose to the olfactory area was 334 µC. We also identified a cutoff point according to the dose to the olfactory epithelium. Olfactory threshold was significantly decreased 2–6 weeks after initiation of therapy, with cumulative local radiation >135 µC (Mann-Whitney U test, P=0.01).

**Conclusion:**

Deterioration in olfactory threshold scores was found at 6 months after initiation of radiation therapy. Provided that these results are reproducible, an evaluation of olfactory functioning in patients with head and neck malignancies using in vivo dosimetry may be useful for determining the optimal dose for patients treated with conformal radiotherapy techniques while avoiding the side effects of radiation.

## Introduction

Olfactory receptors are located within a 5-cm^2^ area of mucosa high in the nasal cavity above the superior turbinate bone. The mucosa is covered by a layer of mucus, and stimulation of receptor cells requires penetration of this aqueous barrier. The receptor cell conducts afferent signals directly through the cribriform plate to the olfactory bulb, where it synapses with various interneurons which may modulate olfactory discrimination (1).

Radiotherapy (RT) is a common treatment modality for patients with head and neck malignancies, and some tumors have a high response rate to this treatment. Normal tissues are often included in the field of radiation and complications due to this treatment are not uncommon (2). Because the nose lies within the field of radiotherapy of the head and neck, the olfactory fibers and olfactory receptors may be affected by radiation. This is relevant because normal olfactory function is important in daily life. Patients with olfactory disorders complain of difficulties associated with cooking (73%), mood changes (68%), decreased appetite (56%), eating of spoiled food (50%), insufficient perception of their own body odor (41%), and burning of food (30%) (3). The inability of an individual with a disabled olfactory function to detect a gas leak in the kitchen can have catastrophic consequences. 

Several reports have indicated smell and taste changes during radiotherapy, but we are aware of only four prospective studies that have specifically focused on changes in smell perception during radiotherapy (4-7). Ophir et al reported an increased olfactory threshold in all patients by the end of treatment with varying degrees of recovery 3–6 months after cessation of treatment (4). Ho et al observed deterioration of olfactory threshold scores 12 months after radiation, although this was not perceived by the patients themselves (5). However, Hua et al and Hölscher et al found no significant difference in olfactory thresholds (6,7). Hölscher et al hypothesized that the olfactory epithelium is relatively resistant to the effects of radiation, while the olfactory bulb/orbitofrontal cortex is responsible for the observed changes of suprathreshold olfactory function (7). The aim of the current study was to investigate this apparent discrepancy in the results of previous studies investigating changes in smell perception during radiotherapy. To the best of our knowledge, this is the first prospective study to investigate the actual dose delivered to the olfactory area using *in vivo* dosimetry and document its effects on the olfactory threshold scores of patients with head and neck malignancies.

## Materials and Methods

*Patient characteristics*

Sixty-one consecutive patients, undergoing radiotherapy for head and neck malignancies at Razi Hospital of the Guilan University from January 2010 to November 2012, were prospectively included in this study. The study was approved by the Ethics Committee of Guilan University of Medical Sciences. All patients gave written informed consent. Seven patients did not return for assessment after radiation, leaving 54 patients who completed the course of radiotherapy and yielded follow-up assessment data. The malignancies were distributed among the 54 patients as follows: nasopharyngeal cancer, 24; paranasal sinus cancer, 12; other head and neck cancers, 18 (including brain tumor, 9; oropharynx carcinoma, 6; and squamous cell carcinoma of skin, 3). 

Twelve patients had undergone surgery, with a mean interval between surgery and olfactory testing of 34.0±12.6 days. No patients had relevant comorbidities such as rhinosinusitis, liver or renal problems, hyperactivity or hypoactivity of the thyroid gland, diabetes, or neurologic disorders. No concurrent medicines (i.e., nasal decongestants) altering the olfaction of the subjects were permitted.

*Study protocol*

Detailed examination of the nose was performed using rigid nasoendoscopy to screen for nasal pathology. Particular attention was given to the nasal septum and superior meatus area to exclude those individuals with mechanical obstructions blocking the pathway to the olfactory fibers at the cribriform plate. All patients were confirmed to have no other nasal disease, including nasal polyposis in particular. Repeated nasoendoscopy was performed to monitor for recurrence of tumor as well as for screening of nasal disease or mechanical obstruction of the pathway to the olfactory fibers in the nasal fossae.

*Olfactory threshold test*

Olfactory function was tested six times during this study; before (0 weeks), during (2,4 and 6 weeks), and 3 and 6 months after radiotherapy. The test battery consisted of 48 sniff bottles (volume, 125 ml; height, 10.5 cm; diameter of opening, 4 cm; screw-on caps with Teflon lids) (8). The 16 bottles contained n-butanol at different dilutions, with the highest concentration at 4%. Dilutions were prepared in a geometric series (dilution ratio of 1:2) using water as a solvent. The remaining 32 bottles were blanks. Using a triple-forced-choice paradigm, detection thresholds were determined by employing a single staircase method as described by Hummel (9). After the cap was removed, the bottle was presented for 3 s, approximately 2 cm in front of both nostrils of the blindfolded subject. Presentation of the triplets to a subject occurred every 20 s, and the subjects were asked to identify the n-butanol-containing bottle. Testing started at the lowest concentration available, then the concentration was increased until correct detection occurred on two consecutive trials. If an incorrect response was given on any trial, the staircase was moved upward one concentration step. If a correct response was given, the staircase was reversed and subsequently moved downward. The threshold was defined as the mean of the last four out of seven staircase reversal points. The subjects’ scores ranged between 0 and 16. Throughout testing, subjects received no feedback as to the accuracy of their responses. The pathologic olfactory threshold was defined as a score of ≤5; i.e. n-butanol cannot be perceived at dilutions greater than dilution step 5, which is equal to a concentration of 0.25% n-butanol (10).

Monitoring the concentration of n-butanol after repeated usage can prove problematic, and this is a known limitation affecting threshold assessments of this type. Therefore, we used a new test battery set after 200 assessments to ensure the quality of the chemical in the bottles. 

In vivo* dosimetry*

*In vivo* dosimetry was performed using 82 LiF (MCP) chips and a thermoluminescent dosimeter (TLD) system. Details of the procedure are provided in a previous paper (11). Using nasal endoscopy, the chips were located above the middle turbinate and fixed with Gelfoam before radiotherapy. The average ratio of measured-to-expected entrance dose was 0.99±0.04.

*Statistical analyses*

Data were analyzed using nonparametric statistics to account for the lack of normal distribution. The primary variable was olfactory threshold score. Comparison of olfactory threshold scores at various timepoints after radiotherapy and between subcategories was performed using a general linear model and the Mann-Whitney U-test, respectively. The statistical analysis was performed using SPSS version 16.0 (Statistical Package for the Social Sciences, Chicago, USA). A two-tailed P<0.05 was considered to indicate statistical significance. All data were reported as median and interquartile range (IQR) for descriptive purposes. 

## Results

Follow-up data were obtained for 26 male and 28 female patients, with a median age of 49 years (range, 22–86 years). 

Most patients were in good general condition (Karnofsky performance status >70%). Six patients (10.7%) were current smokers, and none had a history of excessive alcohol consumption. Twenty-two patients had no cervical lymph node secondary cancer, while N1 disease was present in 24 patients and N2 disease in eight patients. 

No patient had evidence of distant metastasis on presentation, and none had a relapse during follow-up. Chemotherapy, in most cases simultaneous, was part of the therapeutic concept in 44.4% of the patients.

In most patients (36/54), the RT was administered at a dose of 2 Gy once a day, five times each week. The total RT period ranged from 27–48 days (median, 35 days). A conventional radiation technique was used in this study. Off-cord reductions were performed at 40–44 Gy in 20–22 fractions. Radiotherapy was performed with a ^60^Co teletherapy machine (Theratron Phoenix). Most patients received a total radiation dose of 40 Gy in 20 fractions (mean, 50.1 Gy; range, 36–66 Gy; IQR, 40–60 Gy). The planning was based on a simulation CT in most patients. As described in ICRP Report-86, radiotherapy has the potential for accidental exposures (i.e., errors in device operation, source calibration, and treatment calculation) (12). 

Therefore, we used *in vivo* dosimetry to determine the dose delivered to the olfactory area. The median measured dose was 334 µC (IQR, 162-2068 µC).

The results of pretreatment and follow-up assessments using the olfactory threshold test at the completion of radiotherapy and at 3 and 6 months are presented in (Table.1).

**Table 1:** Median of olfactory threshold scores of patients in the different times after radiotherapy

**Time** ** (weeks)**	**Mean**	**Median**	**IQR**
0	11.7	13	8-15
2	5.8	3	2-10
4	6.2	3	1-12
6	5.8	3	1-12
12	5	3	1-7
24	4	2	1-4

During a course of conventional curative RT, olfactory function becomes measurably impaired. There was a significant difference between the mean olfactory threshold of subjects at various timepoints following radiotherapy (general linear model, P<0.0001). There was no significant difference between the olfactory threshold of subjects according to age, gender, radiation regions, or chemotherapy.

**Fig 1:**
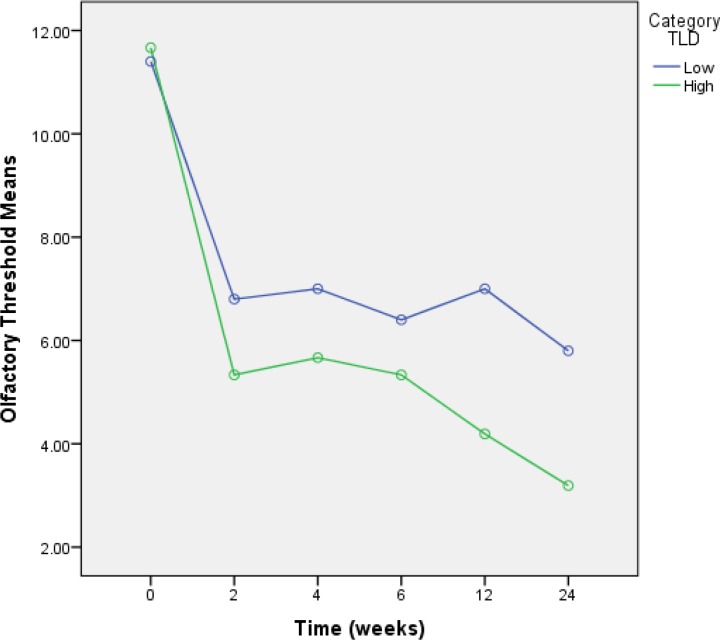
Diagram of olfactory threshold scores of patients in the different times after radiotherapy according to cumulative local radiation

The median cumulative local radiation for olfactory impairment (i.e., olfactory threshold ≤5) was 154 µC (IQR, 58-905 µC). A cutoff point for cumulative local radiation was identified at 135 µC. A significant influence of cumulative local radiation to the olfactory area was confirmed for the olfactory threshold in a nonparametric analysis. The olfactory threshold was significantly decreased 2–6 weeks after initiation of therapy, with cumulative local radiation >135 µC (Mann-Whitney U test, P=0.01) (Table. 2 , Fig. 1).

**Table 2:** Median of olfactory threshold scores from patients in the different times after radiotherapy with regard to cumulative local radiation

**Time (week)**	**Low** *	**High** *
0	10	13
2	4	3
4	4	2.5
6	5	2
12	7	2
24	4	2

* Low and high mean ≤135 µC and >135 µC, respectively

## Discussion

For numerous tumors, including nasopharyngeal or maxillary carcinomas, pituitary adenomas, or sarcomas of the base of skull, RT is an essential part of the therapeutic concept. Although radiation with substantial doses to the olfactory epithelium is inevitable under these conditions, impairment of olfactory function as a side effect of radiotherapy has rarely been reported. The aim of this prospective study was to measure the degree and time course of changes in olfactory function among patients receiving radiotherapy for tumors of the head and neck region. Olfactory thresholds were assessed biweekly during radiotherapy. The patients were followed up for 6 months. 

Although few prospective studies have been performed, several case reports are published relating to the deterioration in olfactory threshold, odor identification, and odor discrimination following radiotherapy. As early as 1975, Schaupp and Krull retrospectively analyzed olfactory function in 19 patients for up to 15 years after radiotherapy (13). These researchers found no abnormal olfactory thresholds after radiotherapy to the maxillary and orbital regions compared with the values of a previously measured ‘normal’ population. Five patients were followed during radiotherapy and were found to have an increased olfactory threshold, with values returning to the initial level 6–8 weeks after radiotherapy.

In a similar prospective study, Ophir et al followed 12 patients with nasopharyngeal carcinoma or pituitary adenoma for up to 6 months after radiotherapy. Radiation doses to the olfactory area were between 18 and 28 Gy (4). Olfactory function significantly decreased during radiotherapy, but varying degrees of recovery were noted in most patients 3–6 months after treatment.

Ho et al measured olfactory threshold, odor identification, and odor discrimination in 48 patients. They used the ‘Sniffin–Sticks’ test within the first 6 months after treatment for nasopharyngeal carcinoma. A mostly negative result was found in this prospective study. Only 12 months after radiotherapy was a significant deterioration described for olfactory thresholds (5).

Hua et al compared three matched groups (healthy subjects, pre-therapeutic, and post-therapeutic patients with nasopharyngeal carcinoma) and showed significant deficits in suprathreshold olfactory function. They noted that the defect of absolute olfactory sensitivity was unable to fully account for higher-order olfactory functional difficulties (6).

The largest study reported was published in the Chinese language only (14). In this study, 100 patients with nasopharyngeal carcinoma were followed for up to 36 months. The smell acuity of enrolled patients decreased sharply 3 months after therapy, but varying degrees of recovery were noted after 6 and 12 months. In general the olfactory acuity of patients decreased after radiotherapy, and did not recover fully. 

Hölscher et al evaluated olfactory function in 44 patients (7), 25 of whom were followed for 12 months. Patients were divided into two groups according to the dose to the olfactory epithelium: median 62.2 Gy (OLF group) and median 5.9 Gy (non-OLF group). This study showed no significant changes in olfactory thresholds; however a significant impairment of odor discrimination or odor identification was noted in the OLF group. The authors hypothesized that the olfactory epithelium is relatively resistant against the effects of radiation and that the observed changes are due to the effects of radiotherapy on the olfactory bulb/orbitofrontal cortex. 

Bramerson et al investigated sense of smell using odor-detection sensitivity and olfactory identification tests (15). Seventy-one patients were assessed before radiotherapy and followed up to 20 months. The authors showed that radiation dose was related to decline in the olfactory function.

Leyrer et al performed a dose-volume histogram analysis in 20 patients with gliomas (16). The patients completed a questionnaire relating to taste and smell disturbances at baseline and at Weeks 3 and 6. Ten of 20 patients reported experiencing some degree of smell disturbance.

Overall, results from previous studies are controversial. While some studies suggested recovery of olfactory function after a few months after radiotherapy, we detected no recovery up to 6 months. As shown in this study, despite the narrow range of total radiation dose, the dose to the olfactory region is highly variable (IQR, 162–2068 µC). This variability may be the cause of the contradictory results of previous studies. There is also a possibility that the in low-radiation doses to the olfactory area (according to *in vivo* dosimetry), changes in the olfactory threshold is due to sensory perception. Another possible reason for deterioration of the olfactory threshold during radiation may be mucosal reactions. Radiotherapy produces mucosal changes in the nasal cavity, such as swelling and mucositis, which could affect the perception of olfactory stimuli (17). Landis et al assumed that the threshold test predominantly reflects peripheral olfactory processes (18). Alterations of the cell cycle and depletion of the basal cells of the olfactory epithelium ultimately result in a confluent mucositis. Damage to the Bowman’s glands in the olfactory mucosa results in massive changes in the environment of the olfactory receptor neurons (4). The regenerative capacities of the olfactory epithelium cause the olfactory function to exhibit recovery after a few months following radiation therapy. At higher radiation doses to the olfactory area (according to in vivo dosimetry), these changes may be associated with nerve damage. Similar to the development of other neurologic changes that have been observed following radiotherapy, the changes in olfaction had a delayed onset of 1 year after radiation. 

## Conclusion

We suggest that evaluation of olfactory functioning in patients with head and neck malignancies using in vivo dosimetry might be useful for determining the optimal dose for patients treated with conformal radiotherapy techniques while avoiding side effects of radiation. Therefore, we advocate initiation of well-designed multicenter clinical trials to investigate this assumption. Further, the possibility of further recovery or deterioration in olfactory function with time after radiation is still unknown, and this issue needs further clarification because of the short follow-up period adopted in the present study.
